# Integrative machine learning and multi-omics framework identifies shared biomarkers for rheumatoid arthritis and ulcerative colitis

**DOI:** 10.1371/journal.pone.0336243

**Published:** 2025-11-10

**Authors:** Meili Liu, Jun Ge, Lei Guo, Zhengzheng Wu, Zimo Cheng, Zenggen Liu, Yi Liu

**Affiliations:** 1 Hubei Provincial Key Laboratory for Chinese Medicine Resources and Chinese Medicine Chemistry, School of Pharmacy, Hubei University of Chinese Medicine, Wuhan, China; 2 Hubei Shizhen Laboratory, Wuhan, China; 3 Wuhan Britain-China School, Wuhan, China; Wenzhou Medical University, CHINA

## Abstract

**Background:**

Rheumatoid arthritis (RA) and ulcerative colitis (UC) are chronic inflammatory diseases with shared immune pathologies but limited common diagnostic biomarkers, which hinders the development of targeted therapies.

**Methods:**

Public gene expression datasets were analyzed to identify differentially expressed genes (DEGs) common to both RA and UC. Functional enrichment and immune infiltration analyses revealed dysregulated pathways. A comprehensive machine learning framework that incorporated 12 algorithms and cross-validation was applied to screen for robust diagnostic biomarkers. Further, RA- and UC-related molecular subtypes were delineated, and the relationship between these shared biomarkers and immune infiltration characteristics was explored. Key findings were validated using single-cell RNA sequencing (scRNA-seq) of UC tissue to localize gene expression and qRT-PCR in cell models mimicking RA and UC.

**Results:**

Analysis identified 19 shared DEGs, with functional enrichment analysis highlighting IL-17 signaling. Machine learning prioritized four key biomarkers (DUOX2, IDO1, NPY1R, SELL) with high diagnostic performance. scRNA-seq localized these genes predominantly to a pro-inflammatory “Macrophage-High” subpopulation and revealed VEGF-mediated crosstalk with endothelial cells. qRT-PCR confirmed significant expression changes of IDO1 and NPY1R in both RA-like and UC-like inflammation models.

**Conclusion:**

This integrative approach identifies DUOX2, IDO1, NPY1R, and SELL as shared RA-UC biomarkers potentially linked to macrophage-driven inflammation and VEGF signaling. These findings provide insights into the common pathogenesis and potential targets for dual-disease diagnostics and therapeutics.

## 1. Introduction

UC, a chronic inflammatory bowel disease (IBD), is clinically characterized by abdominal pain, bloody diarrhea, and weight loss. Its pathogenesis is attributed to intestinal epithelial barrier dysfunction, microbiota dysbiosis, and aberrant immune activation [1,2]. Current therapeutic strategies prioritize endoscopic mucosal healing through the use of immunomodulators, such as mesalazine and JAK inhibitors, as well as biologics [3]. RA, a systemic autoimmune disorder characterized by synovial inflammation and joint destruction [4], shares striking immunopathological parallels with UC. Both diseases exhibit dysregulated IL-17 signaling [5], neutrophil-dominated inflammation [6], and disrupted microbiota-immune crosstalk [7]. Elevated pro-inflammatory cytokines (TNF-α, IL-6) and activated macrophage/T-cell populations further highlight common pathogenic axes [8–10]. Notably, Mendelian randomization studies reveal genetic overlap [11], suggesting shared molecular drivers. However, systematic identification of convergent biomarkers remains elusive, impeding the development of dual-disease diagnostic tools and targeted therapies.

Traditional diagnostic approaches for UC rely on clinical manifestations, such as rectal bleeding, and nonspecific biomarkers including CRP and fecal calprotectin [4,12]. However, these methods have limited sensitivity in the early stages of the disease. While omics technologies have identified disease-specific signatures, the global rise in UC incidence and associated long-term cancer risk underscore the need for better diagnostic methods. Furthermore, studies indicate that RA is more common in IBD patients, and IBD patients have a higher susceptibility to RA. Previous studies on biomarkers for RA-related UC are few. This led to the use of an integrated multi – omics strategy to explore RA-related UC biomarkers. RNA-seq data analysis isn’t precise in reflecting cellular heterogeneity and may conceal differences between cells. By combining bulk RNA-seq data analysis with scRNA-seq data analysis, the study aimed to overcome the limitation of bulk sequencing in masking cellular heterogeneity, allowing for the accurate analysis of transcriptional features within specific cell subpopulations. This enabled the uncovering of unique gene expression patterns, the detection of rare cell types, and the identification of cell-specific molecular markers. In turn, this improved our understanding of cell functions and the mechanisms of UC.

## 2. Methods

### 2.1 Data collection

The gene expression profiling data of ulcerative colitis (GSE3365, GSE87466) and rheumatoid arthritis (GSE55235, GSE55457, GSE77298) were acquired from Gene Expression Omnibus (GEO, http://www.ncbi.nlm.nih.gov/geo). GSE3365 and GSE87466 were used as test sets for UC, while GSE55457 and GSE77298 were used as test sets for RA, to identify the intersecting genes associated with both UC and RA. The combined datasets of GSE3365 and GSE87466 were utilized as the external training set for UC, and the combined datasets of GSE55235, GSE55457, and GSE77298 were used as the external training set for RA, to validate the expression levels and diagnostic performance of the intersecting genes. These datasets are described in S1 File.

### 2.2 Processing of data

The raw datasets underwent rigorous quality control, including the removal of low-quality samples and genes with low expression. The gene expression data of UC and RA were merged separately. The “ComBat” function in the “sva” R package was used to effectively eliminate the observed batch effect. Principal component analysis (PCA) was used to assess the effectiveness of data correction. The analysis aimed to compare the differences in data quality before and after batch removal between the UC and RA groups.

### 2.3 Differential gene expression analysis

The “limma” package was used to analyze the UC and RA datasets for differentially expressed genes (DEGs), selecting those with an adjusted *P*-value (FDR) < 0.05 and |log2 FC| > 1 as statistically significant. Visualization of these DEGs via heatmaps and volcano plots was done in R (4.4.1) using the “ggplot2” package. Common genes among DEGs from the two disease datasets were then extracted and visualized using a Venn diagram. Finally, to explore the interrelation among the common genes, a protein-protein interaction (PPI) network was constructed using STRING (https://cn.string-db.org/).

### 2.4 Enrichment analysis of the common genes

To unravel the biological roles and molecular mechanisms of the shared genes, the “clusterProfiler” package was leveraged to conduct comprehensive Gene Ontology (GO), Kyoto Encyclopedia of Genes and Genomes (KEGG) and Disease Ontology (DO) analyses on these genes. Subsequently, the “enrichplot” package was used to explore the biological functions and signaling pathways, and “ggplot2” was employed to visualize the results.

### 2.5 Machine learning model construction

In-depth analysis of the data was performed using machine learning algorithms. Initially, the raw data was preprocessed by removing missing values and outliers, followed by Z-score normalization to adjust the mean of each feature to 0 and the standard deviation to 1, thereby eliminating the impact of different feature scales. Subsequently, the dataset was randomly divided into a training set (70%) and a testing set (30%). During the model training phase, various machine learning algorithms were employed to evaluate their performance: lasso, Ridge, Elastic Net (Enet), Stepglm, XGBoost, Random Forest (RF), plsRglm, GBM, LDA, glmBoost, Naive Bayes, and SVM. These models were trained, and their hyperparameters were optimized through 10-fold cross-validation. In the model evaluation phase, the testing set was used to calculate the AUC (Area Under the Curve) value for each model (with a threshold set at 0.7) to assess classification performance. The model with the higher AUC value was selected as the optimal model.The Delong test was used to screen for algorithm combinations that are statistically reliable (*p < 0.05*). The corresponding R code is provided in S2 File.

### 2.6 Consistency clustering and immune infiltration analysis

To explore the shared immune characteristics between RA and UC, the ‘GSEABase’ and ‘GSVA’ packages were used to quantify 24 immune cell types in the samples of both diseases using single-sample gene set enrichment analysis (ssGSEA). Subsequently, CIBERSORT was applied to the ssGSEA results to determine the relative abundance of these immune cells within the samples. To better understand the complexity of the diseases, the “ConsensusClusterPlus” package was used to perform unsupervised consensus clustering based on the expression of RA-related UC key genes identified through a PAM clustering approach. Similarly, ssGSEA and CIBERSORT were used to quantify and analyze the 24 types of immune-infiltrating cells in RA and UC sample subgroups, assessing the relative abundance of various immune cells in the disease samples.

### 2.7 ROC diagnostic model construction

Prognostic nomogram models effectively integrate multiple metrics to predict disease onset and progression. From machine learning models, four key genes were identified, and a prognostic nomogram model was constructed based on these genes. “Rms” and “Rmda” package were used to assess disease risk based on these genes. The model’s performance was evaluated with ROC analysis via package. The diagnostic accuracy of these genes was confirmed in both internal and external datasets.

### 2.8 UMAP plot for cell clustering

Single-cell transcriptomes from the GSE214695 dataset were processed using Seurat v4.0. After rigorous quality control (mitochondrial gene percentage <20%, nFeature >500), the data were normalized via SCTransform and subjected to UMAP clustering (resolution = 0.8). Cell types were annotated based on marker genes from the CellMarker database. Additionally, six algorithms (AUCell, UCell, singscore, ssGSEA, JASMINE, and viper) from the “irGSEA” package were used to score gene sets in the scRNA-seq datasets [13].

### 2.9 Trajectory analysis

To explore the differentiation trajectories within the macrophage cell type, the cytoTRACE algorithm was employed alongside Monocle R (2.24.0) and Slingshot (2.6.0) software tools. Utilizing the DDRTree algorithm, trajectories were reconstructed and cell lineages were inferred through minimum spanning trees (MSTs) to monitor the developmental progression along the identified paths.

### 2.10 Cell communication

The CellChat R package was used to analyze gene expression data and explore variations in potential cell-cell communication networks. Employing the conventional CellChat pipeline, the default CellChatDB was relied on for ligand-receptor interactions. By identifying overexpressed ligands or receptors within specific cell groups, cell type-specific interactions were inferred.

### 2.11 Construction of cell line model and real-time quantitative PCR analysis

To validate bioinformatics analysis results and explore their potential roles in the pathological processes of RA and UC, the study selected four key genes (IDO1, NPY1R, DUOX2, SELL) for qRT-PCR validation. The MH7A, Caco2, and RAW264.7 cell lines were cultured in DMEM medium (G4511; Servicebio, China) containing 10% fetal bovine serum (FBS, Servicebio, China). To establish an in vitro RA model, MH7A cells were stimulated with 5 μg/mL lipopolysaccharide (LPS, GC205009, Servicebio, China) for 24 hours, and then collected for analysis. To simulate inflammation-related macrophages in colitis, Caco2 cells were pretreated with 20 μg/mL LPS for 24 hours, and then co-cultured with RAW264.7 macrophages for 24 hours using Transwell® inserts (WG3415, Servicebio, China; pore size 3 μm), with Caco2 cells in the upper chamber and RAW264.7 cells in the lower chamber to mimic the interaction between intestinal epithelium and macrophages. After co-culturing, cells were collected for analysis. Total RNA was extracted from the samples using a total RNA extraction kit (Servicebio, China). One microgram of total RNA was reverse transcribed into cDNA using the SweScript All-in-One RT SuperMix for qPCR (One-Step gDNA Remover) (Servicebio, China). DNA amplification and detection were performed using the 2 × Universal Blue SYBR Green qPCR Master Mix (Servicebio, China) on the StepOnePlus™ real-time PCR system (Thermo Fisher Scientific). The data were analyzed using the 2^(-ΔΔCT) method, the data were normalized using GAPDH as the internal reference gene and all experiments were repeated independently three times (specific primers are listed in S2 Table). The experiment was conducted under conditions that comply with biosafety regulations.

### 2.12 Statistical analysis

Data preprocessing and visualization were performed using R languages. The statistical differences between two groups were evaluated using the Wilcoxon rank-sum test and Student’s t-test, while differences between multiple groups were tested using the ANOVA method. A multiple correction was applied, and a p-value of < 0.05 was considered statistically significant, with significance levels defined as * p < 0.05, **p < 0.01, and ***p < 0.001.

## 3. Results

### 3.1 Data processing

**Fig 1:**
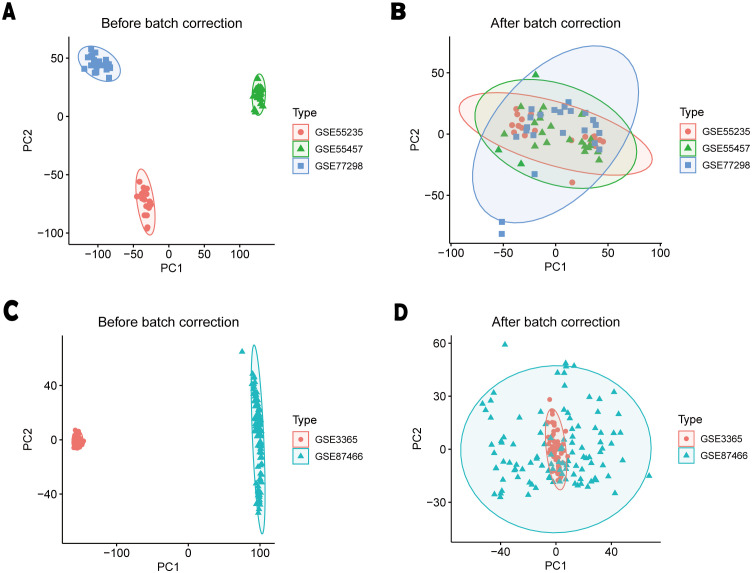
Integration of datasets. **(A)** PCA of the datasets GSE55235, GSE55457, and GSE77298 after batch effect correction;**(B)** PCA of the integrated RA dataset after batch effect correction;**(C)** PCA of the datasets GSE3365 and GSE87466 after batch effect correction;**(D)** PCA of the integrated UC dataset after batch effect correction.

PCA showed the successful integration of transcriptomic datasets. For RA, this yielded a processed cohort consisting of 39 RA cases and 27 control samples (Fig 1A, 1B). Similarly, UC and control datasets were combined after batch correction, creating a normalized validation cohort of 113 UC cases and 63 controls (Fig 1C, 1D). These steps significantly reduced batch effects.

### 3.2 Common genes

**Fig 2:**
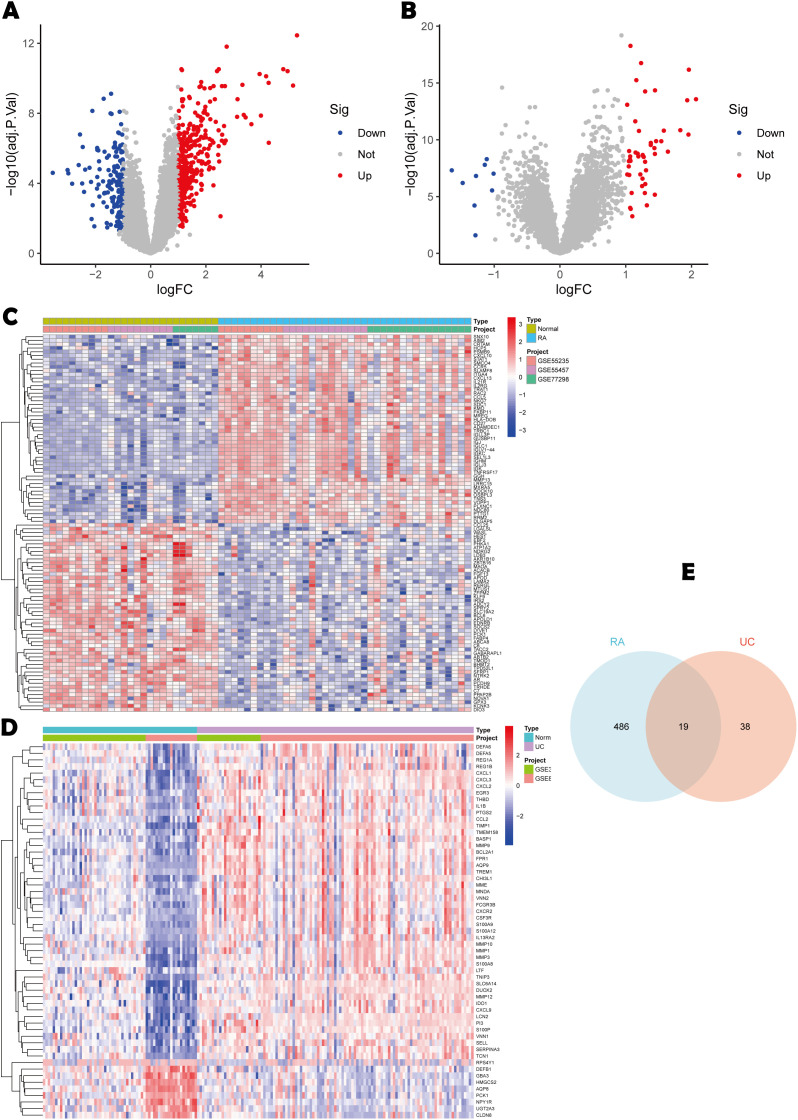
Identification of DEGs. **(A)** Volcano plot of DEGs between RA and healthy controls;**(B)** Volcano plot of DEGs between UC and healthy controls;**(C)** Heatmap of DEGs between RA and healthy controls;**(D)** Heatmap of DEGs between UC and healthy controls;**(E)** The 19 shared DEGs between UC and RA.

Differential expression analysis revealed distinct transcriptional landscapes in RA and UC. For the RA cohort, 505 DEGs were identified, with 344 up-regulated and 161 down-regulated (Fig 2A, 2C). In the UC cohort, 57 DEGs were identified, including 48 up-regulated and 9 down-regulated (Fig 2B, 2D). Further analysis found 19 DEGs common to both UC and RA (Fig 2E).

### 3.3 Functional enrichment of common genes

**Fig 3:**
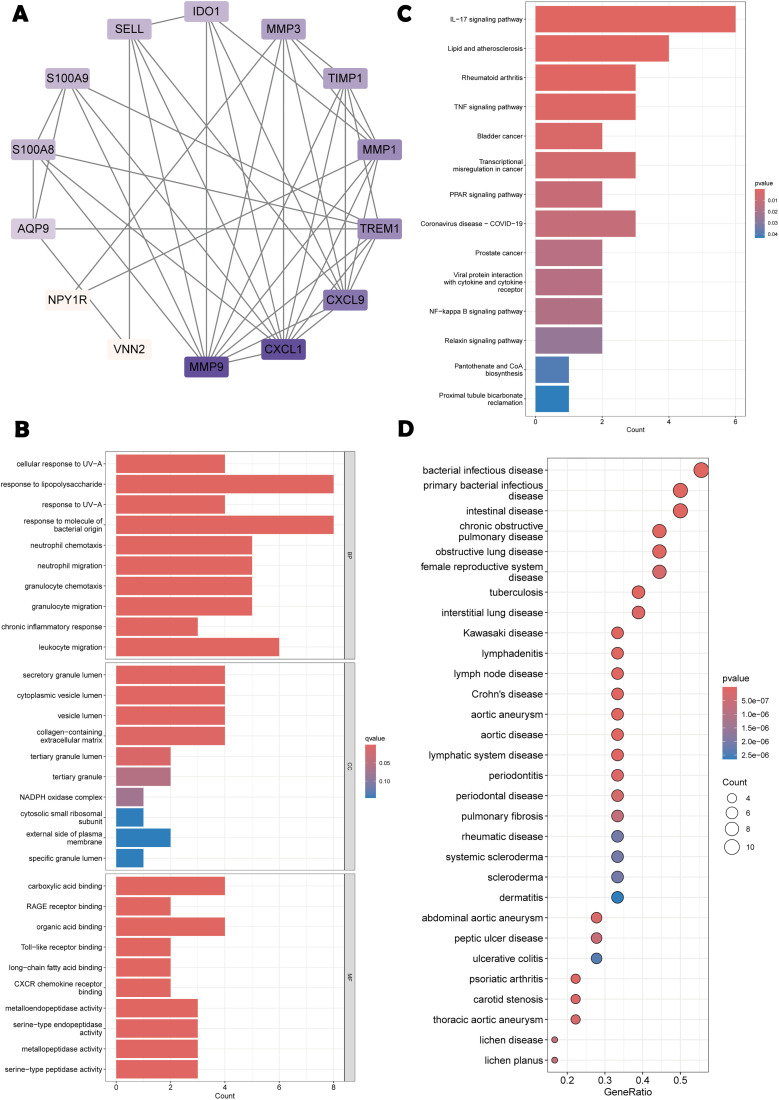
PPI network analysis and enrichment analysis. **(A)** PPI network of key genes constructed by STRING;**(B)** Bar plot of GO pathway enrichment analysis results;**(C)** KEGG pathway enrichment analysis results;**(D)** DO pathway enrichment analysis results.

PPI network analysis of the 19 common genes revealed functional connectivity (Fig 3A). GO enrichment indicated roles in response to lipopolysaccharide, bacterial molecules, and leukocyte migration (Fig 3B). KEGG analysis showed significant enrichment in IL-17 signaling, lipids, and atherosclerosis (Fig 3C). DO analysis highlighted involvement in bacterial infectious diseases, particularly intestinal ones (Fig 3D).

### 3.4 Immune infiltration analysis of common genes

**Fig 4:**
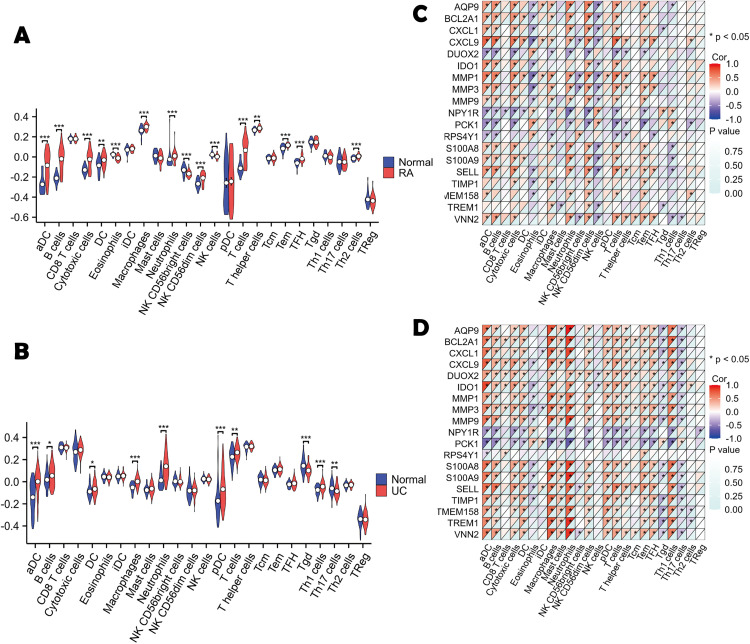
Immune infiltration analysis. **(A)** Boxplot of immune cell abundance between RA and controls;**(B)** Boxplot of immune cell abundance between UC and controls;**(C)** Heatmap of immune cell infiltration in RA;**(D)** Heatmap of immune cell infiltration in UC. ***P < 0.001; **P < 0.01; *P < 0.05; ns, not significant.

Immune infiltration analysis revealed distinct cellular landscapes in each disease. Compared to controls, RA samples showed significant enrichment of activated dendritic cells (aDC), B cells, cytotoxic cells, eosinophils, macrophages, neutrophils, NK subsets (CD56bright, CD56dim), and T cell populations (Tem, TFH, Th2) (Fig 4A). In UC, aDC, macrophages, neutrophils, plasmacytoid dendritic cells (pDC), and Th1 cells were elevated, while Tgd cells were reduced (Fig 4B). Heatmaps (Fig 4C-4D) showed abundance changes of common genes in RA and UC immune cells, closely linked to immune activation, inflammation, tolerance, and suppression.

### 3.5 Machine models

**Fig 5:**
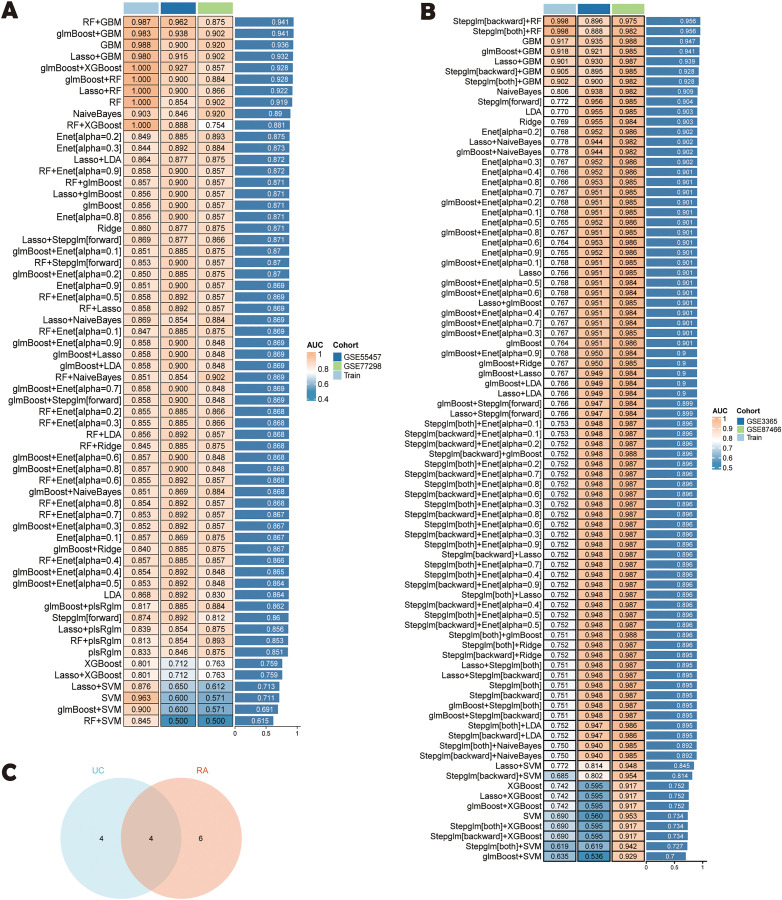
Machine learning models. **(A)** Machine learning model for RA;**(B)** Machine learning model for UC; **(C)** Four genes at the intersection of RA and UC models.

A comprehensive machine learning framework incorporating 12 algorithms with 10-fold cross-validation identified the most robust diagnostic model based on 19 shared genes (Fig 5A, 5B). Specifically, for RA, the combination of glmBoost and GBM algorithms produced the best results, achieving an AUC of 0.983 in the training cohort and 0.938, 0.902 in the validation cohort, with an average AUC of 0.941. Ten key genes (MMP1, DUOX2, CXCL9, RPS4Y1, AQP9, PCK1, VNN2, SELL, IDO1, and NPY1R) were identified using the glmBoost + GBM algorithm. For UC, the combination of Stepglm[backward] and RF algorithms produced the best results, achieving an AUC of 0.998 in the training cohort and 0.896, 0.975 in the validation cohort, with an average AUC of 0.96. Eight key genes (MMP9, TIMP1, CXCL1, IDO1, NPY1R, SELL, BCL2A1, and DUOX2) were identified through the Stepglm[backward] + RF algorithm. Fig 5C shows four genes (DUOX2, IDO1, NPY1R, SELL) that are common to both RA and UC. See S1 Table for a comprehensive comparison of all models.

### 3.6 Immune infiltration analysis of key genes

**Fig 6:**
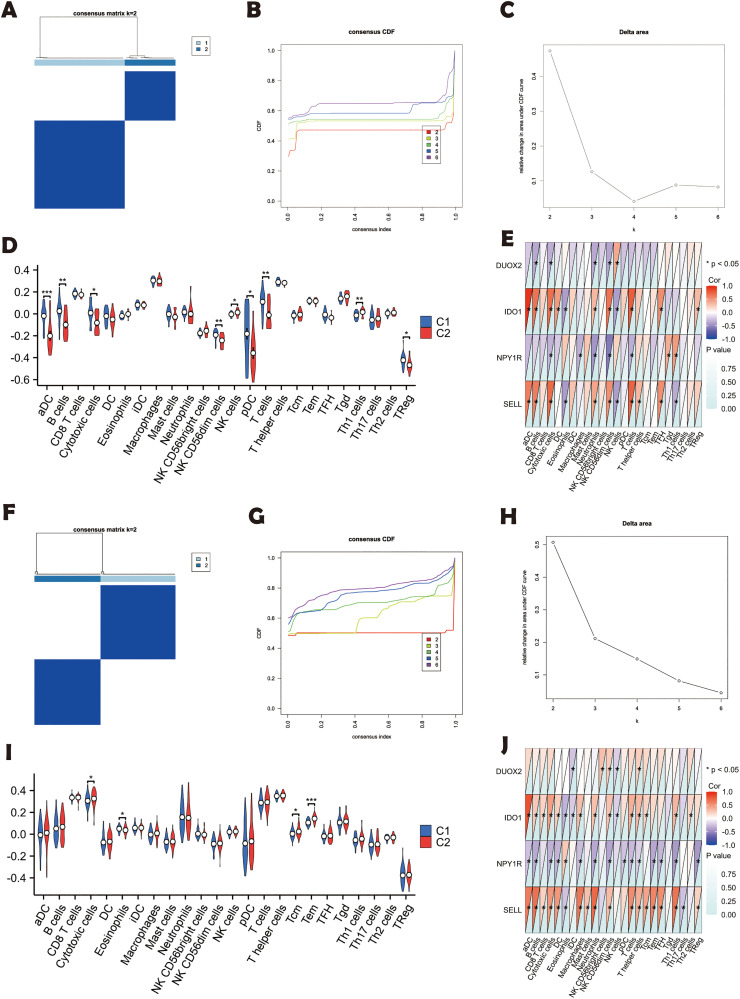
Disease subtype based on key genes. **(A)** Consistency clustering heatmap for RA; **(B)** CDF plot of RA consistency clustering results; **(C)** Delta Area plot of RA consistency clustering results; **(D)** Boxplot of differentially expressed gene-related expression in RA subtypes; **(E)** Heatmap of differentially expressed gene-related expression in RA subtypes; **(F)** Consistency clustering heatmap for UC; **(G)** CDF plot of UC consistency clustering results; **(H)** Delta Area plot of UC consistency clustering results; **(I)** Boxplot of differentially expressed gene-related expression in UC subtypes; **(J)** Heatmap of differentially expressed gene-related expression in UC subtypes. ***P < 0.001; **P < 0.01; *P < 0.05; ns, not significant.

Unsupervised consensus clustering of 4 DEGs partitioned RA (n = 39) and UC (n = 113) cohorts into two subtypes (k = 2, Fig 6A, 6F), with a significant difference area change from k = 2 to k = 6 (Fig 6B-6C, 6G-6H). RA subtype C2 exhibited increased aDC, B cells, cytotoxic cells, NK subsets, pDC, and T cells, alongside reduced Th1 and Treg cells (Fig 6D-6E). UC subtype C2 displayed elevated cytotoxic cells, eosinophils, and memory T cells (Tcm, Tem; Fig 6I-6J). These disease-specific immune dysregulations highlight actionable targets for precision immunotherapy.

### 3.7 ROC diagnostic results

**Fig 7:**
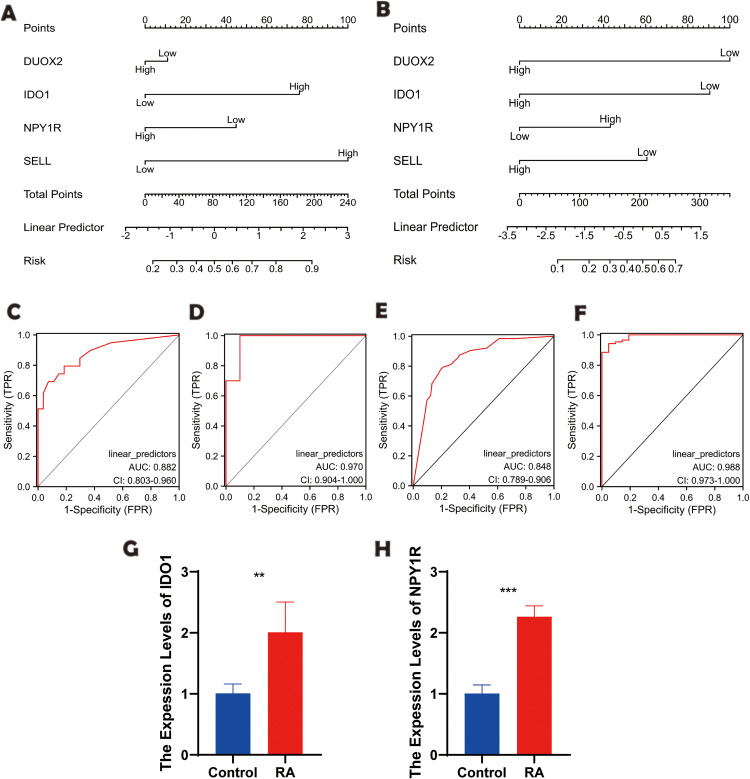
Evaluation of the diagnostic performance of key genes and expression detection of key genes in MH7A cell lines and LPS-induced MH7A cell lines. **(A)** Nomogram model for RA based on four key genes; **(B)** Nomogram model for UC based on four key genes; **(C-D)** ROC curve analysis of four key genes in the RA training set and test set; **(E-F)** ROC curve analysis of four key genes in the UC training set and test set; **(G-H)** The mRNA expression levels of IDO1 and NPY1R validated by qRT-PCR in MH7A cell lines and LPS-induced MH7A cell lines. Data are presented as mean ± SD. Statistical significance was determined using a two-tailed Student’s t-test, with * p < 0.05, ** p < 0.01, and *** p < 0.001 compared to control MH7A cell lines (n = 3).

ROC curves and a diagnostic model were constructed based on the four genes (Fig 7 A-7F). The reliability of these genes was assessed using ROC analysis (S2 Fig).

### 3.8 qRT-PCR key genes in vitro of RA

The expression levels of these four key genes were assessed in the MH7A cell line and the LPS-induced MH7A cell line. In the LPS-induced MH7A cell line, the mRNA expression levels of IDO1 and NPY1R were significantly upregulated compared to the normal MH7A cell line (Fig 7G-7H and S1 Fig).

### 3.9 UMAP plot for cell clustering and trajectory analysis

**Fig 8:**
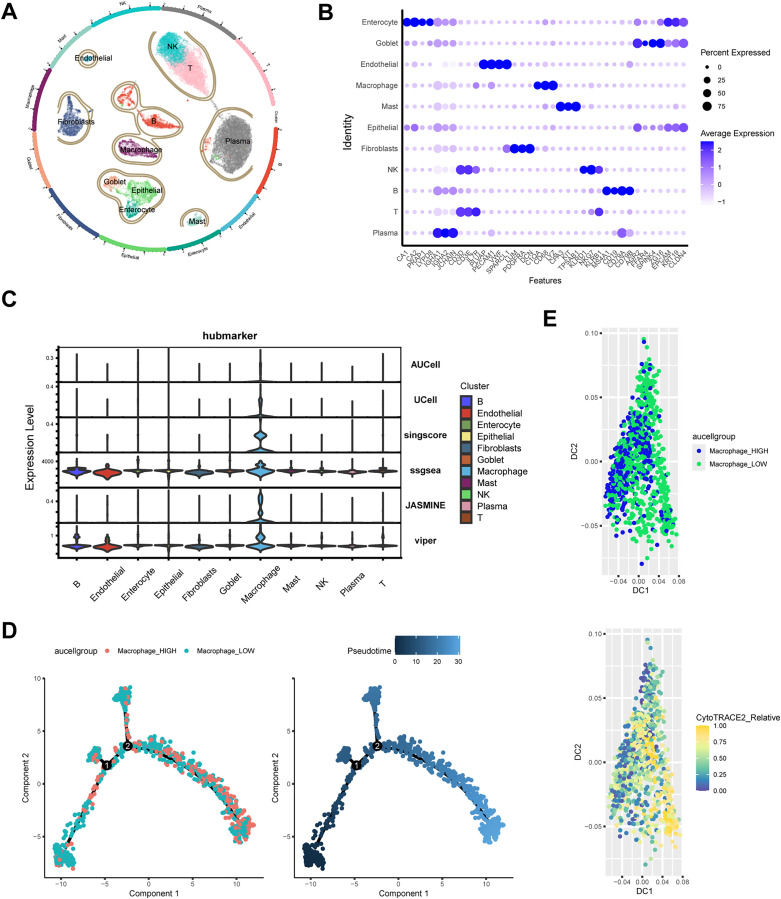
UMAP Plot for Cell Clustering and trajectory analysis. **(A)** UMAP Plot for Cell Clustering. **(B)** Expression levels for selected marker genes of each celltype. **(C)** Violin Plot for hubmarker Expression in each cell type. **(D/E)** Pseudo-time and trajectory analyses of macrophages.

Single-cell transcriptomic analysis of UC samples (GSE214695) identified 11 distinct cell clusters (Fig 8A). Marker gene expression patterns across these populations provided a comprehensive view of cellular heterogeneity (Fig 8B). Combining six gene set scoring algorithms, the key biomarkers were found to be mainly localized in macrophage populations (Fig 8C). Total macrophages were divided into Macrophage-High and Macrophage-Low groups based on singscore. Trajectory analysis showed that the proportion of the Macrophage-High group was lower in the early stages of development and increased in the later stages (Fig 8D). CytoTRACE analysis further defined the developmental order and starting point of macrophages, revealing that the Macrophage-High group primarily resided in the terminal stages of development (Fig 8E).

### 3.10 Cellchat results presentation

**Fig 9:**
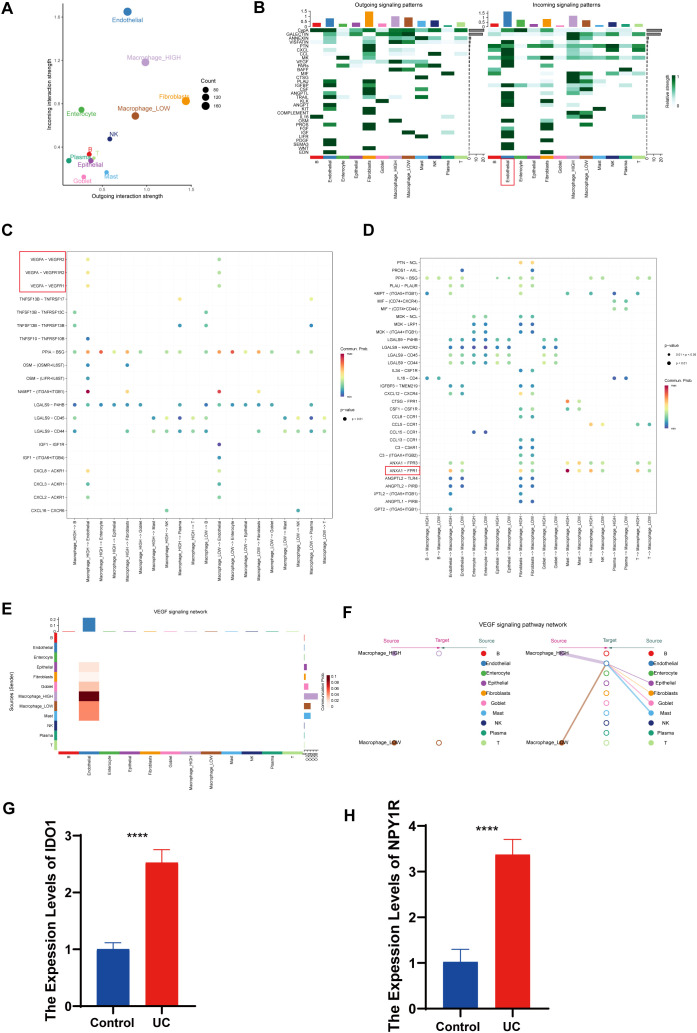
Cellchat results presentation and expression detections of key gene in RAW264. cell lines and co-cultured RAW264.7 cell lines. **(A)** Number or strength of cell population interactions. **(B)** Heatmap of cellular communication. Heatmap showing afferent and efferent signal intensities of all cell interactions. **(C)** Outcoming contribution bubble plots showing the expression of cellular communication patterns. **(D)** incoming contribution bubble plots showing the expression of cellular communication patterns. **(E)** Interaction of cells in the VEGF signaling pathway shown by heatmap. **(F)**Macrophage as a receiver interaction ligand diagram. Hierarchical diagram of macrophage celltype interacting with other cells in the VEGF signaling pathway. **(G-H)** qRT-PCR analysis showing the mRNA expression levels of IDO1 and NPY1R in RAW264.7 cell lines and co-cultured RAW264.7 cell lines. Data are presented as mean ± SD. Statistical significance was determined using a two-tailed Student’s t-test, with * p < 0.05, ** p < 0.01, and *** p < 0.001 compared to control RAW264.7 cell lines (n = 3).

High macrophages exhibited a higher total volume of intercellular communication compared to Low macrophages (Fig 9A). Heatmaps depicted signal intensity of intercellular interactions (Fig 9B). Fig 9C and 9D further investigated the ligand-receptor interactions between various cell types and both High and Low macrophages within UC intestinal mucosal tissues. It was found that High macrophages communicated with endothelial cells through VEGFA-VEGFR2, VEGFA-VEGFR1R2 and VEGFA-VEGFR2 signaling pathways. In terms of signal reception, endothelial, fibroblasts, and NK cells communicated more frequently with High macrophages via ANXA1-FPR1 ligand-receptors. Pathway visualization revealed mechanisms of VEGF signaling (Fig 9E). Analysis of the quantity and intensity of cellular communication revealed that High-macrophages had significantly enhanced interactions with other cells, suggesting a potential synergistic relationship between them (Fig 9F). These findings align with immune cell infiltration results, underscoring a potential link between hub genes and immune cells, particularly Macrophage cells, in the pathogenesis of RA and UC.

### 3.11 qRT-PCR key genes in vitro of UC

Expression levels of these four key genes were also assessed in vitro using the RAW264.7 cell line and a co-culture model. In co-cultured RAW264.7 cells, mRNA expression levels of IDO1 and NPY1R were significantly upregulated compared to the normal RAW264.7 cell line (Fig 9G-9H and S1 Fig).

## 4. Discussion

The global incidence of immune-mediated inflammatory diseases (IMIDs), particularly ulcerative colitis (UC) and rheumatoid arthritis (RA), has surged in recent decades, imposing substantial clinical and economic burdens [11]. Emerging evidence underscores their shared pathogenesis, including dysregulated IL-17 signaling, microbiota-immune axis disruption, and neutrophil-dominated inflammation [14,15]. Mendelian randomization studies further support their genetic interplay, with overlapping risk loci implicating common immunopathogenic mechanisms [16]. Notably, Korsunsky et al [17] identified conserved fibroblast activation states in both diseases, while microbiome studies reveal causal links between gut dysbiosis and systemic inflammation [18]. Recent multi-omics analyses have further highlighted the role of macrophage plasticity in bridging intestinal and joint inflammation, with transcriptional reprogramming driven by epigenetic modifiers such as HDAC3 [19–23]. Despite these advances, the molecular drivers of their comorbidity remain poorly defined, hindering dual-disease therapeutic strategies.

This study integrated a machine learning framework to prioritize four biomarkers (DUOX2, IDO1, NPY1R, SELL) from the 19 shared DEGs between RA and UC. Each of these molecules may play a distinct and important role in inflammatory processes.

IDO1 is a key immunomodulatory enzyme. It may contribute to immune tolerance, primarily through the TGF-β pathway during antigen sensitization [24,25]. It also might promote angiogenesis and IL-6 production through GCN2-mediated stress responses [26]. This contributes to chronic inflammation and offers a potential therapeutic target for autoimmune conditions [27]. DUOX2 is critical for mucosal oxidative defense and microbiome homeostasis. Dysfunction of DUOX2 amplifies IL-17C-driven inflammation in response to Gram-negative bacteria [28,29]. In UC, the expression of DUOX2 is increased. This disrupts intestinal barrier integrity and promotes dysbiosis [30]. DUOX2 may also influence mucin expression, further affecting barrier function and immune regulation [31]. NPY1R is a typical G-protein-coupled receptor (GPCR) that has been extensively studied for its role in regulating intestinal inflammation. It modulates pro-inflammatory signaling by interacting with the pro-inflammatory cytokine TNF [32]. NPY1R regulates diverse processes, including cell proliferation, osteoblast differentiation, and pain perception. This highlights its broad role in chronic inflammation and bone remodeling in RA [33–35]. SELL (CD62L) is an adhesion molecule that facilitates leukocyte rolling and trans-endothelial migration (TEM) [36]. It enables neutrophil infiltration into inflamed tissues via interactions with PECAM-1 [37]. SELL is implicated in immune cell aggregation in synovial and intestinal mucosa, making it a key player in both UC and RA [38].

Notably, single-cell RNA sequencing confirmed the co-localization of these four biomarkers within a specific macrophage subpopulation (Macrophage-High cluster). This subpopulation dynamically expands as the disease progresses, suggesting it may be a key driver of pro-inflammatory responses. This finding provides crucial cellular insights into the mechanisms underlying disease progression.

Macrophage functional polarization is a core mechanism regulating inflammation. In the classical paradigm, macrophages are divided into pro-inflammatory M1 and anti-inflammatory/repair-oriented M2 types [21,39,40]. M1 macrophages are characterized by high secretion of tumor necrosis factor-α (TNF-α), interleukin-1β (IL-1β), and interleukin-12 (IL-12), and elevated expression of surface markers such as CD86 and NOS2, which collectively amplify local inflammatory responses. In contrast, M2 macrophages mainly secrete interleukin-10 (IL-10) and transforming growth factor-β (TGF-β), and express markers like CD206, CD163, and Arg1, which are critical for inflammation resolution, tissue regeneration, and maintenance of immune homeostasis.

Within this framework, the Macrophage-High subpopulation is closer to the M1 phenotype. Its increase during disease progression correlates with the enrichment of M1 macrophages in the acute inflammatory phase. Additionally, the high expression of the IDO1 gene in this subpopulation. IDO1 is involved in immune regulation and is often upregulated in M1 macrophages—further supports its pro-inflammatory functional tendency. However, our data suggest that the Macrophage-High subpopulation may not represent the canonical M1 subset. Its signature genes are associated not only with inflammation but also with mucosal microenvironment adaptation and inflammatory signaling modulation, indicating non-classical M1 features.

Beyond intrinsic polarization states, cell-cell communication analysis revealed that VEGF-mediated crosstalk between macrophages and endothelial cells may represent a convergent pathogenic axis. In rheumatoid arthritis (RA), synovial macrophages overexpress VEGF-A, promoting angiogenesis and SELL-dependent leukocyte extravasation [37,41]. Conversely, in ulcerative colitis (UC), macrophage-derived VEGF compromises intestinal microvascular integrity, facilitating neutrophil recruitment [42]. CellChat analysis supports this mechanism by demonstrating that macrophage-derived VEGF ligands activate endothelial FLT1/KDR receptors. This finding aligns with preclinical evidence showing that VEGF blockade improves both conditions [43,44], highlighting a shared therapeutic target for these comorbid diseases.

Several limitations should be considered. First, although multiple public datasets were integrated, the overall sample size is still limited, which may affect the statistical power and limit the generalizability of the study conclusions. Second, although the machine learning model performed well in internal cross-validation, the complete predictive model has not yet been externally validated in a prospective cohort from a completely different source. There may still be a risk that the model may be overfitted to the characteristics of the current dataset, and its clinical translation potential needs further verification. Third, while the bioinformatics evidence from single-cell data supports the central role of macrophages in the mechanism, the precise causal roles of the identified biomarkers need further functional validation through cell-specific knockout or knockdown models.

## 5. Conclusion

This study establishes a novel integrative framework for identifying shared biomarkers in UC and RA. DUOX2, IDO1, NPY1R, and SELL were identified as key diagnostic biomarkers showing strong performance in machine learning models. Mechanistically, these genes are linked to dysregulated inflammatory and immune pathways, particularly IL-17 signaling and VEGF-mediated macrophage-endothelial crosstalk, as revealed by functional enrichment and single-cell transcriptomics. Although only IDO1 and NPY1R showed significant trends in qRT-PCR validation, further studies are needed to fully elucidate the roles of DUOX2 and SELL in these specific contexts. Collectively, DUOX2, IDO1, NPY1R, and SELL represent promising candidates for developing diagnostic tools and targeted therapies for both RA and UC.

## Supporting information

S1 File Basic information of GEO datasets used in the study. (DOCX)

S2 File R code. (DOCX)

S1 Fig Expression detections of SELL and DUOX2. (PDF)

S2 Fig ROC curves of biomarkers on the external validation cohort. (PDF)

S1 Table All models comparison table. (XLSX)

S2 Table The gene primer sequences. (XLSX)
