# Supplementary material for: Integrative machine learning and multi-omics framework identifies shared biomarkers for rheumatoid arthritis and ulcerative colitis
Source: PLoS One. 2025 Nov 10;20(11):e0336243. doi: 10.1371/journal.pone.0336243 (PMC12599921; doi:10.1371/journal.pone.0336243)
Supplement: S1 File — (DOCX) [file pone.0336243.s001.docx]

**Table 1**. Basic information of GEO datasets used in the study.

| GSE series | Organism | Samples | Source types | Platform |
| --- | --- | --- | --- | --- |
| GSE3365 | Homo sapiens | 26 UC patients and 42 normal controls | PBMC | GPL96 |
| GSE87466 | Homo sapiens | 87 UC patients and 21 normal controls | Mucosal biopsies | GPL13158 |
| GSE55235 | Homo sapiens | 10 RA patients and 10 normal controls | Synovial tisssue | GPL96 |
| GSE55457 | Homo sapiens | 13 RA patients and 10 normal controls | Synovial membrane | GPL96 |
| GSE77298 | Homo sapiens | 16 RA patients and 7 normal controls | Synovial biopsies | GPL570 |
| GSE93272 | Homo sapiens | 232 RA patients and 43 normal controls | Whole blood | GPL570 |
| GSE48958 | Homo sapiens | 13 UC patients and 8 normal controls | Mucosal | GPL6244 |
| GSE214695 | Homo sapiens | 6 UC patients and 6 normal controls | Colonic mucosa | GPL18573 |

UC, ulcerative colitis; RA, rheumatoid arthritis; PBMC, Peripheral blood mononuclear cell.

GSE3365 [**GEO Accession viewer**](https://www.ncbi.nlm.nih.gov/geo/query/acc.cgi?acc=GSE3365)

GSE87466 [GEO Accession viewer](https://www.ncbi.nlm.nih.gov/geo/query/acc.cgi?acc=GSE87466)

GSE55235 [GEO Accession viewer](https://www.ncbi.nlm.nih.gov/geo/query/acc.cgi?acc=GSE55235)

GSE55457 [GEO Accession viewer](https://www.ncbi.nlm.nih.gov/geo/query/acc.cgi?acc=GSE55457)

GSE77298 [GEO Accession viewer](https://www.ncbi.nlm.nih.gov/geo/query/acc.cgi?acc=GSE77298)

GSE214695 [GEO Accession viewer](https://www.ncbi.nlm.nih.gov/geo/query/acc.cgi?acc=GSE214695)

GSE93272 [GEO Accession viewer](https://www.ncbi.nlm.nih.gov/geo/query/acc.cgi?acc=GSE93272)

GSE48958 [GEO Accession viewer](https://www.ncbi.nlm.nih.gov/geo/query/acc.cgi?acc=GSE48958)
