# Supplementary material for: Integrative machine learning and multi-omics framework identifies shared biomarkers for rheumatoid arthritis and ulcerative colitis
Source: PLoS One. 2025 Nov 10;20(11):e0336243. doi: 10.1371/journal.pone.0336243 (PMC12599921; doi:10.1371/journal.pone.0336243)
Supplement: S2 File — (DOCX) [file pone.0336243.s002.docx]

Detailed information can be found in the code section (<https://anonymous.4open.science/r/codes-051C/>).
