# Supplementary material for: Integrative machine learning and multi-omics framework identifies shared biomarkers for rheumatoid arthritis and ulcerative colitis
Source: PLoS One. 2025 Nov 10;20(11):e0336243. doi: 10.1371/journal.pone.0336243 (PMC12599921; doi:10.1371/journal.pone.0336243)
Supplement: S1 Fig — (PDF) [file pone.0336243.s003.pdf]

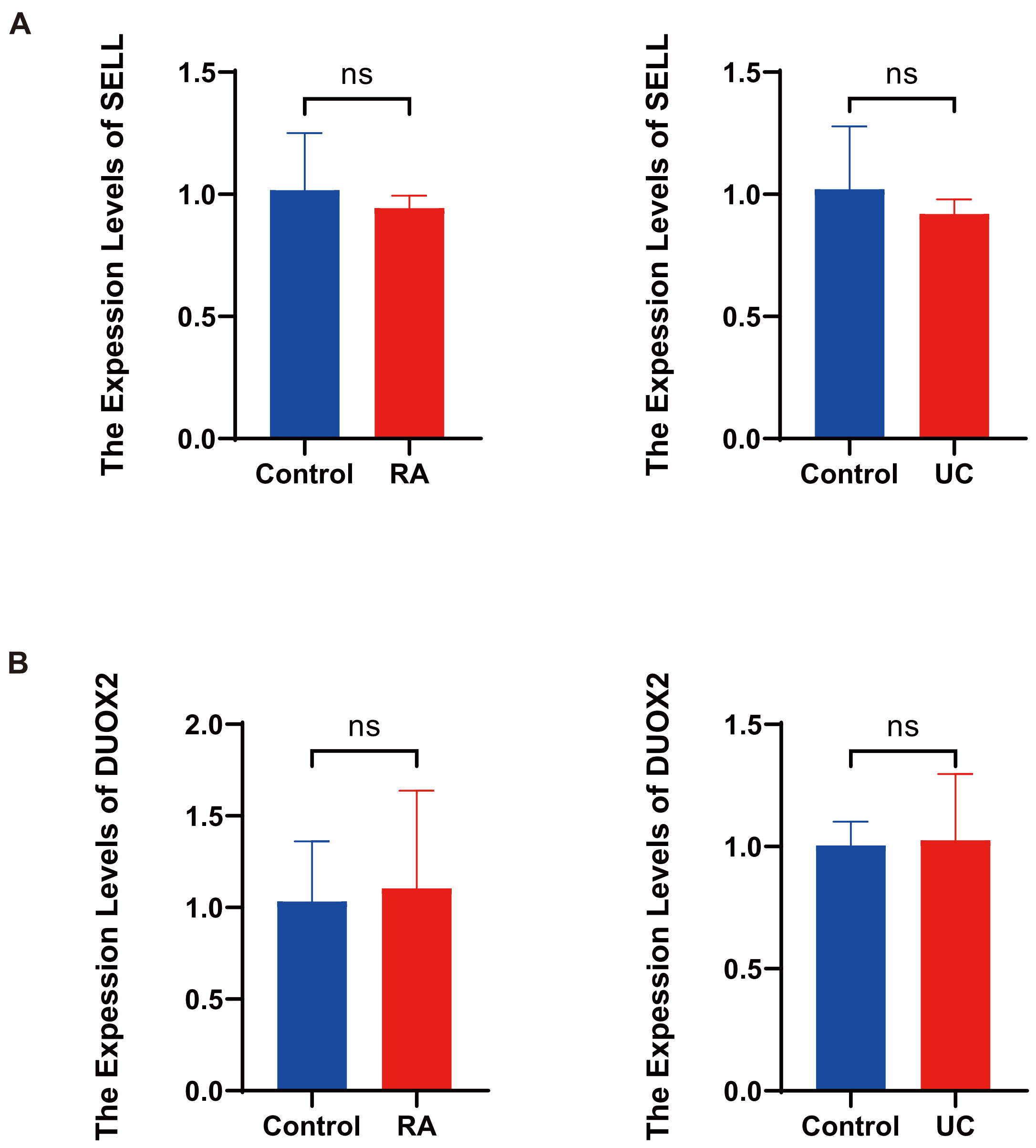

A
The Expression Levels of SELL
1.5
1.0
0.5
0.0
ns
Control
RA
UC
B
The Expession Levels of DUOX2
2.0
1.5
1.0
0.5
0.0
ns
Control
RA
UC
