# Supplementary figures and images for: Integrative machine learning and multi-omics framework identifies shared biomarkers for rheumatoid arthritis and ulcerative colitis
Source: PLoS One. 2025 Nov 10;20(11):e0336243. doi: 10.1371/journal.pone.0336243 (PMC12599921; doi:10.1371/journal.pone.0336243)

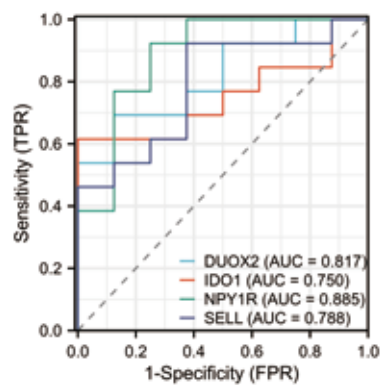

UC: GSE48958

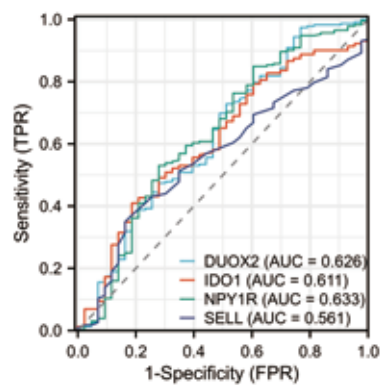

GSE93272

Supplement: S2 Fig — (PDF) [file pone.0336243.s004.pdf]
